# Pain Trajectories and Factors Associated with Repeated Greater Occipital Nerve Block in Post-Dural Puncture Headache: A Retrospective Observational Cohort Study

**DOI:** 10.3390/jcm15187079

**Published:** 2026-09-12

**Authors:** Sinan Pektaş, Çağatay Küçükbingöz

**Affiliations:** 1 Division of Algology (Pain Medicine), Department of Anesthesiology and Reanimation, Faculty of Medicine, Muğla Sıtkı Koçman University, 48000 Muğla, Türkiye; 2 Division of Pain Medicine, Department of Anesthesiology and Reanimation, Adana City Training and Research Hospital, 01370 Adana, Türkiye; ckbingoz.md@gmail.com

**Keywords:** post-dural puncture headache, greater occipital nerve block, pain trajectory, repeated nerve block, epidural blood patch, retrospective cohort study, observational study, penalised logistic regression

## Abstract

**Background**: Epidural blood patch (EBP) remains the standard procedural treatment for post-dural puncture headache (PDPH) persisting after conservative care; whereas, the evidence for greater occipital nerve block (GONB) is limited. This study describes pain trajectories during GONB-based management and factors associated with repeated GONB. **Methods**: Single-centre retrospective observational cohort of all consecutive patients treated for PDPH between January 2016 and January 2018. Eligible patients met the current ICHD-3 criteria after spinal anaesthesia, had a Numerical Rating Scale (NRS) score ≥ 4 and had persistent symptoms after ≥24 h of conservative management. Bilateral landmark-guided GONB (2.5 mL bupivacaine HCl 5 mg/mL per side) was repeated when the NRS remained ≥4 at a 24 h assessment, so group membership reflected the response to treatment. **Results**: Sixty-two patients were analysed (mean age 33.9 ± 15.1 years; 64.5% women). NRS decreased over seven days (Friedman χ^2^ = 262.6, *p* < 0.001; Kendall’s W = 0.847); on day 7, 57 patients (91.9%; 95% CI 82.5–96.5%) had absent-to-mild pain. Thirty-eight patients (61.3%; 95% CI 48.8–72.4%) received one block. In an exploratory Firth penalised model, baseline NRS (OR 2.41, 95% CI 1.55–4.15) and orthopaedic surgery (OR 5.02, 95% CI 1.36–21.56) were associated with repeated GONB. No GONB-related complications and no EBP were documented. **Conclusions**: NRS scores decreased during a strategy comprising one or more GONB sessions with concomitant conservative care, but these uncontrolled observations cannot separate any effect of GONB from spontaneous recovery, regression to the mean or concomitant treatment. They are hypothesis-generating and require prospective randomised confirmation.

## 1. Introduction

Post-dural puncture headache (PDPH) is a headache attributed to dural puncture, whether intentional, as in spinal anaesthesia or diagnostic lumbar puncture, or unintentional, as during epidural catheter placement. In the current International Classification of Headache Disorders, third edition (ICHD-3), the diagnosis requires a headache that develops within five days of the dural puncture and is not better accounted for by another diagnosis; an orthostatic pattern is characteristic but is no longer obligatory [1]. Contemporary guidance describes the underlying process as loss of cerebrospinal fluid (CSF) through the dural defect, with a reduction in intracranial CSF volume and/or pressure, downward displacement of intracranial structures, traction on pain-sensitive intracranial tissues and compensatory intracranial vasodilatation [2,3]. PDPH should therefore not be equated with a measured reduction in CSF pressure alone; volumetric studies indicate that the change in CSF volume relates more closely to symptoms than any single pressure measurement [4]. The risk of PDPH is influenced by needle gauge and tip design and by the number of puncture attempts, and the reported incidence after spinal anaesthesia varies widely with the population studied and the needle used [2,5].

Despite this frequency, awareness and management of PDPH remain suboptimal. A recent survey found that a substantial proportion of clinicians who perform lumbar puncture were not familiar with current diagnostic and treatment standards [6]. Pregnancy, younger age and female sex are recognised risk factors [2].

PDPH is frequently self-limiting, but its course is neither rapid nor uniform. Classic observational data reported spontaneous resolution in 59% of patients within four days and in 80% within one week after spinal anaesthesia with cutting needles [7]; whereas, in a randomised trial of conservatively managed moderate-to-severe PDPH, 86% of patients still reported headache on day 7 and more than half rated the residual symptoms as moderate or severe [8]. A minority of patients, particularly after unintentional dural puncture in obstetric practice, experience headaches lasting months [9]. During the symptomatic period, upright activity, maternal–newborn care, breastfeeding and discharge planning may all be limited. Early postoperative mobilisation is an established component of enhanced recovery pathways and is associated with a shorter length of stay [10,11]; this general literature should not, however, be read as evidence that PDPH itself causes thromboembolism, atelectasis, pneumonia or muscle deconditioning. None of these outcomes were measured in the present cohort.

Initial management of PDPH is symptom-directed and typically includes patient education, hydration, simple analgesics such as paracetamol and non-steroidal anti-inflammatory drugs (NSAIDs), and short-term caffeine or other adjuvants [2,3]. Bed rest may provide temporary postural relief but is not recommended as routine prolonged treatment, because it has not been shown to alter the course of the condition [2]. For clinically significant PDPH that persists despite conservative measures and limits daily activity, the epidural blood patch (EBP) remains the standard and most effective procedural intervention [2,8]. Transient back pain is common after EBP; whereas, infection, neurological injury, arachnoiditis and subdural haematoma are rare [2,3]. These uncommon complications do not diminish the established role of EBP; rather, they provide a reason to evaluate less invasive symptomatic options in appropriately selected patients.

The greater occipital nerve (GON) arises predominantly from the dorsal ramus of the second cervical spinal nerve and provides sensory innervation to the occipital scalp. GON block (GONB) has an accepted role in several primary and secondary headache disorders, including migraine, cervicogenic headache and occipital neuralgia [12]; interventions targeting the GON, comprising local anaesthetic block and, in refractory cases, pulsed radiofrequency, have been reported in other headache phenotypes [13,14]. Two anatomical observations make this approach of particular interest in PDPH rather than merely plausible by analogy with primary headache. First, the dura of the posterior cranial fossa receives a substantial part of its sensory innervation from the upper cervical dorsal root ganglia (C1–C3) rather than from the trigeminal ganglion, as shown by retrograde tracer studies and confirmed in subsequent anatomical reviews [15,16]. Second, these upper cervical afferents and trigeminal afferents converge on shared second-order neurons in the trigeminocervical complex, and experimental stimulation of the GON alters the excitability of those neurons [17,18,19]. A block of the GON therefore acts on part of the same afferent pool that conveys nociception from the caudal dura, which offers a mechanistic rationale beyond cutaneous anaesthesia alone. This nevertheless remains a hypothesis in PDPH, because it has not been tested directly in this condition, and observations in other headache phenotypes, whether from small comparative series or isolated case reports [13,14,20], cannot establish efficacy. Clinical studies of GONB in PDPH, including randomised trials, have reported encouraging results [21,22,23,24,25,26,27], but they differ in population, block level and technique, local anaesthetic and adjuvants, and comparator and outcome definition, which limits direct comparison between them. Current multisociety guidance accepts that GONB may be considered in selected patients while continuing to emphasise EBP for persistent, function-limiting symptoms [2]. The position of GONB within the PDPH treatment pathway therefore remains uncertain.

The primary objective of this study was to describe the seven-day trajectory of NRS pain scores during a GONB-based management strategy in patients with moderate-to-severe PDPH that persisted after conservative treatment following spinal anaesthesia. Secondary objectives were to describe how many GONB sessions patients received and when they were performed, to examine whether baseline pain intensity and surgical category were associated with receiving repeated blocks, and to describe the clinical pathway, including rescue treatment and documented complications.

## 2. Materials and Methods

### 2.1. Ethical Approval and Study Design

This study was conducted and documented in compliance with the Strengthening the Reporting of Observational Studies in Epidemiology (STROBE) standards [28]. It was a single-centre retrospective observational cohort study that was approved by Van Training and Research Hospital’s Institutional Ethics Committee (date: 15 March 2018; decision no: 2018/05). All procedures were conducted in accordance with the ethical principles outlined in the Declaration of Helsinki. Due to the retrospective nature of the data review, written research consent was not obtained from everyone. However, as part of the institution’s regular clinical practice, all patients sign a general informed permission form at the point of service, clearly allowing the use of clinical data for educational and scientific reasons; this procedure was fully followed by all patients included in this study. Prior to analysis, all data were anonymised, and personal identifiers were eliminated from the dataset.

**Figure 1:**
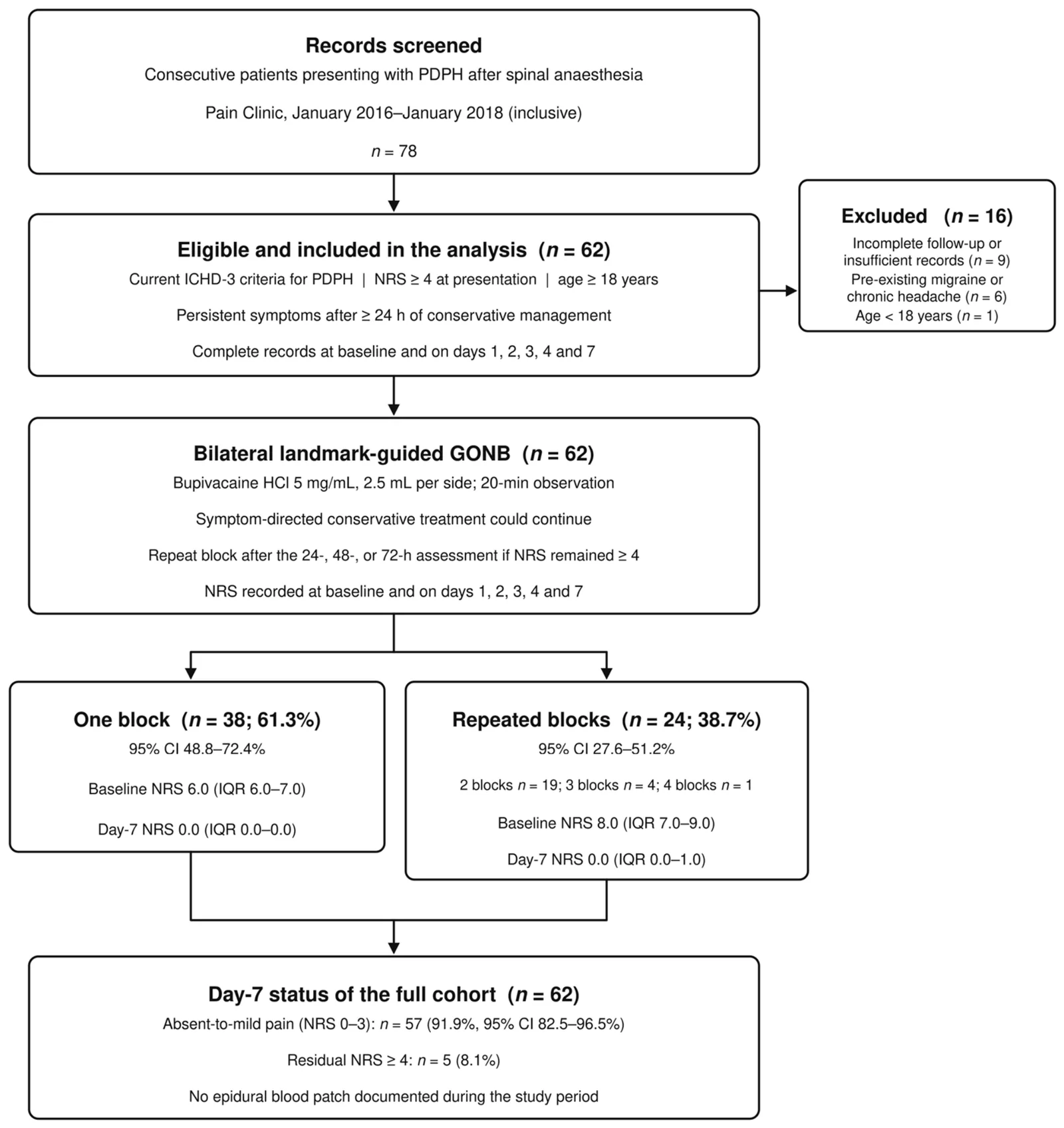
Patient flow diagram (STROBE). The single study period, January 2016 through January 2018 (inclusive), is the one used throughout the manuscript. PDPH: post-dural puncture headache; GONB: greater occipital nerve block; NRS: Numerical Rating Scale; IQR: interquartile range; CI: confidence interval; ICHD-3: International Classification of Headache Disorders, 3rd edition.

All consecutive patients who presented with PDPH and received GONB at the Pain Clinic of Van Training and Research Hospital from January 2016 through January 2018 (inclusive) were screened. This single period is used throughout this manuscript, in Figure 1 and in all tables. Of the 78 patients screened, 62 met the prespecified eligibility criteria and were analysed.

### 2.2. Eligibility Criteria

The inclusion criteria were (1) PDPH after spinal anaesthesia that could be classified according to the current ICHD-3 criteria [1]; (2) an NRS pain score of ≥4 at presentation; (3) persistent symptoms after at least 24 h of conservative management comprising hydration and at least two analgesic or adjuvant medications; (4) age ≥ 18 years; and (5) available clinical records at baseline and on days 1, 2, 3, 4 and 7. All eligible records were drawn from the single study period defined in Section 2.1; no data from outside this period were used.

The exclusion criteria were (1) a history of dural puncture during epidural anaesthesia (only patients who had undergone spinal anaesthesia were eligible); (2) a prior diagnosis of chronic headache, migraine or occipital neuralgia; (3) incomplete clinical documentation or missing data at any scheduled assessment point; (4) age under 18 years; and (5) lack of a signed informed consent form.

### 2.3. Diagnosis, Assessment, and Follow-Up

For the present analysis, the recorded diagnoses were reassessed against the current ICHD-3 criteria: a dural puncture had occurred, the headache developed within five days of that puncture, and no other diagnosis better accounted for the presentation [1]. The clinical records also documented characteristic worsening of the headache in an upright position with improvement when supine, although this feature is not mandatory in the current classification. In each case, a pain specialist evaluated the temporal relationship to the spinal anaesthetic, the associated symptoms, the neurological examination and alternative causes of postoperative headache. Pain intensity was recorded on an 11-point NRS (0 = no pain; 10 = worst imaginable pain) and categorised as absent-to-mild (0–3), moderate (4–7) or severe (8–10). The NRS score was documented before the initial block and on days 1, 2, 3, 4 and 7, and all patients were followed in the outpatient pain clinic for one week as part of routine clinical practice.

The institutional standard protocol, which requires the use of a 25-gauge Quincke (cutting-tip) spinal needle as the default instrument across all surgical specialties, was followed in all spinal anaesthetic procedures. During the whole trial period (January 2016–January 2018), this protocol was consistently followed.

Before GONB, all patients received the institutional symptom-directed regimen for at least 24 h: oral and/or intravenous fluids totalling 2000–3500 mL/day together with at least two agents selected from paracetamol, caffeine, an NSAID, theophylline and aminophylline. The choice of agents was based on the patient’s age, body weight, comorbidities and breastfeeding status. Failure of conservative management was defined operationally as a persistent NRS score of ≥4 after this period. Bed rest formed part of local practice at the time and was used for temporary postural symptom relief; it is not presented here as a disease-modifying treatment, and current guidance does not support prolonged routine bed rest [2]. Because medication was individualised and the retrospective records did not consistently preserve the agent-specific dose, route and duration, or analgesic consumption after the block, these exposures could not be standardised or analysed and are acknowledged as a source of residual confounding. Symptom-directed conservative medication could be continued after GONB at the discretion of the treating physician.

### 2.4. The Intervention

All procedures were performed by a single pain specialist using a standardised anatomical landmark-guided technique. Intravenous access was established before the procedure, and non-invasive blood pressure monitoring, pulse oximetry and three-lead ECG monitoring were maintained throughout. With the patient seated and the neck slightly flexed, 2.5 mL of bupivacaine HCl (Buvasin®, VEM İlaç San. ve Tic. A.Ş., Çerkezköy, Tekirdağ, Türkiye), 5 mg/mL; 12.5 mg per side) was injected bilaterally through a 25-gauge needle at the medial third of the line connecting the mastoid process and the external occipital protuberance, immediately medial to the occipital artery [22]. Local compression was applied for 2 min, and all patients were observed for 20 min, with the sensory effect verified by means of pinprick. At each 24 h assessment, an NRS score of <4 was regarded as adequate analgesia for the purposes of the protocol. If the NRS score remained ≥4, the same block was repeated after that assessment; second, third and fourth sessions were therefore administered approximately 24, 48 and 72 h after the initial block. Because this rule was applied prospectively in clinical practice, membership of the single-block and repeated-block groups was determined by the response to treatment rather than by any baseline characteristic.

### 2.5. Statistical Analysis

All analyses were performed with SPSS version 22.0 (IBM Corp., Armonk, NY, USA) and independently verified in Python 3.11 (SciPy 1.17). The distribution of continuous variables was assessed with the Shapiro–Wilk test. Non-normally distributed continuous variables are reported as the median (interquartile range, IQR: Q1–Q3), categorical variables as *n* (%) and selected proportions with Wilson 95% confidence intervals (CIs). Within-patient change in NRS scores across the six assessments was examined with the Friedman test, with Kendall’s W as the omnibus repeated-measures effect size; each follow-up assessment was compared with the baseline using Wilcoxon signed-rank tests with Bonferroni correction and the matched-pairs rank correlation r as the effect size. The association between baseline NRS score and the total number of sessions was quantified with Spearman’s rho and an approximate Fisher-transformed 95% CI. Comparisons between the single-block and repeated-block groups, and between the caesarean and non-caesarean subgroups, used the Mann–Whitney U test for continuous variables, with the rank-biserial correlation r as the effect size, and the chi-square or Fisher exact test for categorical variables; Cramér’s V summarised the overall association between surgical category and group membership, and a Monte Carlo exact test (200,000 permutations) was added because two expected cell counts were below five. Because membership of these groups was determined by the response to treatment rather than at baseline, all such comparisons are interpreted descriptively and are subject to indication and response bias. In line with proposed guidance for pain research, absolute values of r of 0.10, 0.30 and 0.40 were treated as tentative thresholds for small, medium and large individual-difference effects, with corresponding thresholds of 0.10, 0.30 and 0.70 for standardised group differences and of 1.20, 1.72 and 3.55 for odds ratios (ORs) [29]. Confidence intervals are interpreted as measures of estimation uncertainty and compatibility rather than as significance tests [30]. The specified multivariable model originally included seven covariates for only 24 events (3.4 events per variable), far below the ratio generally recommended for stable estimation [31], and sex was collinear with caesarean delivery because all caesarean patients were women; that model was therefore abandoned. It was replaced by a single prespecified, parsimonious model with two covariates (baseline NRS score and orthopaedic versus other surgery; 12 events per variable) fitted using Firth’s penalised likelihood, which reduces small-sample bias and yields finite estimates in sparse data [32,33]; profile-likelihood 95% CIs and penalised likelihood-ratio *p* values are reported. This analysis is exploratory and is presented for hypothesis generation only. All tests were two-sided, and *p* < 0.05 was regarded as statistically significant.

## 3. Results

### 3.1. Patients Characteristics

All 78 consecutive patients who presented with PDPH during the study period were screened. Sixteen were excluded because of incomplete follow-up or insufficient clinical documentation (*n* = 9), pre-existing migraine or chronic headache (*n* = 6) or age below 18 years (*n* = 1), leaving 62 patients for analysis (Figure 1). The exclusion of nine patients for incomplete records may have introduced selection bias, and no information was available on the subsequent course of the excluded patients.

The demographic and clinical features are presented in Table 1. The mean age was 33.9 ± 15.1 years (range 18–72) and the mean body mass index (BMI) was 25.1 ± 3.6 kg/m^2^. The cohort comprised 40 women (64.5%) and 22 men (35.5%). PDPH occurred predominantly after caesarean section (C/S; *n* = 24, 38.7%), followed by orthopaedic (*n* = 19, 30.6%), urological (*n* = 13, 21.0%) and general surgical (*n* = 6, 9.7%) procedures. Baseline NRS scores did not differ between the surgical categories (Kruskal–Wallis H = 2.610, df = 3, *p* = 0.456).

### 3.2. Pain Trajectory over Time

The median pre-block NRS score was 7.0 (IQR 6.0–8.0); 36 patients (58.1%) had moderate pain and 26 (41.9%) had severe pain, and, by definition, no patient had an NRS score below 4 at baseline. During management with one or more GONB sessions plus concomitant symptom-directed care, the median NRS score was 3.5 (IQR 2.0–4.75) on day 1, 2.0 (IQR 1.0–3.75) on day 2, 1.0 (IQR 0.0–2.75) on day 3, 0.0 (IQR 0.0–2.0) on day 4 and 0.0 (IQR 0.0–0.0) on day 7. Absent-to-mild pain was recorded in 31 patients (50.0%) on day 1, in 46 (74.2%) on day 2 and in 57 (91.9%; 95% CI 82.5–96.5%) on day 7, and no patient was in the severe category from day 3 onwards. Because repeat blocks were administered according to the 24 h response rule, the values recorded from day 2 onwards reflect the management strategy as a whole and cannot be attributed to the initial block. Table 2 and Table 3 present the observed trajectory and the distribution of severity categories.

The distribution of NRS scores changed across the six assessments (Friedman χ^2^ = 262.6, df = 5, *p* < 0.001), with a large omnibus effect size (Kendall’s W = 0.847). Every follow-up assessment differed from baseline after Bonferroni correction (all adjusted *p* < 0.001), with matched-pairs effect sizes of r = 0.60–0.62 (Table 2). This large within-cohort temporal effect describes the change observed during the management period; in the absence of a control group, it does not estimate a treatment effect of GONB, and its magnitude cannot be used to infer causality.

### 3.3. Block Frequency, Correlation, and Group Comparison

Thirty-eight patients received a single GONB session (61.3%; 95% CI 48.8–72.4%), 19 received two (30.6%), four received three (6.5%) and one received four (1.6%); the 24 patients who received repeated blocks represented 38.7% of the cohort (95% CI 27.6–51.2%). Baseline NRS was associated with the total number of sessions (Spearman ρ = 0.556; approximate 95% CI 0.356–0.708; *p* < 0.001), which exceeds the tentative threshold for a large individual-difference effect in pain research [29].

For descriptive purposes the patients were grouped according to whether the 24 h response rule resulted in a single block (*n* = 38) or in repeated blocks (*n* = 24) (Table 4). Age, body mass index and the distribution of sex were similar between the groups (all *p* > 0.30; |r| ≤ 0.16). Surgical category differed overall (χ^2^ = 8.093, df = 3, *p* = 0.044; Monte Carlo exact *p* = 0.042; Cramér’s V = 0.361), with orthopaedic procedures accounting for 50.0% of the repeated-block group and caesarean procedures for 50.0% of the single-block group; no category-specific post hoc comparison was performed, and two expected cell counts were below five. The median baseline NRS was 8.0 (IQR 7.0–9.0) in the repeated-block group and 6.0 (IQR 6.0–7.0) in the single-block group (*p* < 0.001; r = 0.62), and the NRS distributions also differed on days 1 to 4 (r = 0.29–0.72) but not on day 7 (*p* = 0.069). Because group membership was itself determined by the response at 24 h and by subsequent clinical decisions, these trajectories are descriptive: they do not demonstrate distinct biological response phenotypes and they cannot be read as a comparison of treatment effects.

### 3.4. Post-Caesarean Versus Non-Caesarean PDPH: Subgroup Comparison

The 24 patients who had undergone caesarean delivery and the 38 who had undergone non-caesarean surgery were compared (orthopaedic *n* = 19, urological *n* = 13, general surgery *n* = 6) (Table 5). Age, body mass index and baseline NRS score distributions were similar in these subgroups (all *p* > 0.24). Repeated blocks were administered to five of the 24 caesarean patients (20.8%; 95% CI 9.2–40.5%) and to 19 of the 38 non-caesarean patients (50.0%; 95% CI 34.8–65.2%; Fisher exact *p* = 0.032), and the median number of blocks was 1.0 (IQR 1.0–1.0) versus 1.5 (IQR 1.0–2.0), respectively (*p* = 0.015; r = 0.32). The NRS score distribution changed over time within both subgroups (Friedman *p* < 0.001 in each); whereas, no between-subgroup comparison of NRS scores at any follow-up assessment approached significance (all *p* ≥ 0.61; |r| ≤ 0.08), and the proportions with absent-to-mild pain on day 7 were almost identical (91.7% versus 92.1%). Sex could not be compared because all caesarean patients were women; thus, surgical category and sex were structurally confounded in this cohort. These post hoc comparisons were further confounded by the response-based treatment decisions and should not be interpreted as evidence that caesarean patients respond more rapidly, or require fewer blocks, because of the type of surgery.

### 3.5. Exploratory Penalised Multivariable Analysis

The originally planned seven-covariate logistic model could not be estimated in a stable manner: with 24 events, it provided only 3.4 events per variable, produced very wide confidence intervals (for orthopaedic surgery, OR 8.84, 95% CI 1.24–63.04), and included sex, which is collinear with caesarean delivery. It was therefore replaced by a single prespecified two-covariate model estimated using Firth’s penalised likelihood (12 events per variable). In this model, each one-point increase in baseline NRS score (OR 2.41, 95% profile-likelihood CI 1.55–4.15; *p* < 0.001) and orthopaedic surgery compared with all other surgical categories (OR 5.02, 95% CI 1.36–21.56; *p* = 0.015) were associated with the receipt of repeated GONB (Table 6). With the tentative thresholds adopted here [29], these correspond to medium-to-large and large associations, respectively. The penalised estimate for orthopaedic surgery is considerably more conservative than the unpenalised estimate, and its interval is much narrower, but it remains imprecise. Receipt of repeated GONB is a clinician-mediated outcome determined after baseline; these coefficients therefore describe factors associated with the number of procedures actually performed and must not be read as predictors that could be used to individualise treatment.

### 3.6. Clinical Pathway, Rescue Treatment and Safety

No GONB-related complication was documented in any record during the observation period. Adverse events were not, however, recorded prospectively on a structured form, so under-ascertainment of minor or self-limiting events cannot be excluded. Five patients (8.1%) still had an NRS score of ≥4 on day 7. No epidural blood patch was documented for any patient during the study period; whether EBP was formally offered, declined by the patient or judged unnecessary could not be determined reliably from the retrospective records, so the cohort provides no information on the use of rescue EBP. Systematic NRS assessments ended on day 7, and standardised measurements beyond that point were unavailable. Analgesic consumption after the block, time to mobilisation, discharge timing, breastfeeding and patient satisfaction were not recorded in a form that permitted analysis.

## 4. Discussion

This retrospective observational cohort study describes the NRS trajectories observed during a clinical strategy comprising an initial bilateral GONB, response-triggered repeat blocks and concomitant conservative care. NRS scores declined over the seven-day follow-up. Thirty-eight patients received a single block and 24 received repeated procedures; in exploratory analyses, higher baseline pain intensity and orthopaedic surgery were associated with a greater number of sessions. These observations do not establish that GONB was effective. Spontaneous recovery, regression to the mean after selection for NRS scores of ≥4, continued conservative medication and the additional blocks themselves could each have contributed to the later values, and the comparisons between the single-block and repeated-block groups were conditioned on the response to treatment and are therefore subject to indication and response bias.

The findings must be read against the variable natural history of PDPH. Historical and controlled data show that spontaneous recovery occurs over days to weeks but is far from universal within the first week [7,8]. The proportion of patients with absent-to-mild pain on day 7 in this cohort (91.9%) therefore cannot be compared as an efficacy estimate with the outcomes of trials that differ regarding populations, endpoints, co-interventions and treatment allocations. The pathophysiology of PDPH involves CSF loss, a reduction in intracranial CSF volume and/or pressure, downward displacement of intracranial structures, traction on pain-sensitive tissues and compensatory vasodilatation [2,3,4]; convergence within the trigeminocervical complex remains a plausible hypothesis for symptomatic modulation by an occipital block, rather than a demonstrated mechanism in this condition.

A specific anatomical argument makes GONB worth evaluating in PDPH rather than merely plausible by analogy with primary headache. Retrograde tracer work and subsequent anatomical reviews indicate that the dura of the posterior cranial fossa receives a substantial part of its sensory supply from the upper cervical dorsal root ganglia (C1–C3), rather than from the trigeminal ganglion [15,16]. In intracranial hypotension, the loss of CSF volume permits caudal displacement of the brain and brainstem such that traction falls preferentially on these caudal, cervically innervated structures and on the craniocervical junction; this is consistent with the occipital and nuchal localisation and the neck stiffness that frequently accompany PDPH [17]. Because the GON is the dorsal ramus of C2, a block placed at the occiput interrupts part of the same afferent pool. Upper cervical and trigeminal afferents converge in turn on shared second-order neurons in the trigeminocervical complex, where occipital input has been shown experimentally to activate second-order neurons through NMDA-dependent mechanisms and to modulate their responsiveness through GABAergic and glycinergic pathways [18,19]. Two consequences would follow: an immediate reduction in nociceptive traffic reaching the trigeminocervical complex, and a reduction in the sensitisation of those neurons. This may explain why analgesia after GONB has been reported to outlast the pharmacological duration of the local anaesthetic [12]. Neither effect, however, restores CSF volume or seals the dural defect. With this reasoning, GONB would be symptomatic rather than disease-modifying, which is a further reason why it cannot be regarded as a substitute for EBP and why any benefit would be expected to be temporary if the underlying leak persists. The present uncontrolled data can neither confirm nor refute this account, and the mechanism is offered here as a rationale for future controlled study, rather than as an explanation of the trajectories observed.

PDPH can substantially limit upright activity, maternal–newborn care and readiness for discharge. The present dataset, however, did not capture mobilisation, thromboembolic or pulmonary complications, length of stay, breastfeeding or functional recovery, and the general literature supporting early postoperative mobilisation [10,11] should not be treated as evidence about complications directly attributable to PDPH. Similarly, the greater representation of orthopaedic patients among those receiving repeated blocks may reflect baseline pain severity, the surgical context, clinician preference or unmeasured factors; it does not support a proactive multi-session strategy.

Randomised and observational studies have reported improvement after GONB in PDPH [21,22,23,24,25,26,27], and randomised comparisons with other peripheral blocks or injection techniques provide useful clinical context [24,25]. Such studies support continued investigation, but they do not remove the confounding present in this cohort, and differences between obstetric and non-obstetric populations; distal, proximal and bilevel techniques; in the local anaesthetic and adjuvants used; and in outcome definitions preclude direct numerical benchmarking. In particular, the proportion of patients here who required only one block should not be interpreted as a response rate equivalent to the pain-relief outcomes of controlled trials. Differences in technique—for example, the two-level block evaluated by Mahdy et al. [25] compared with the single-level distal block with bupivacaine alone used here—are informative for the design of future trials but cannot explain between-study differences in the absence of direct randomised comparison.

EBP remains the standard procedural treatment for clinically significant PDPH that persists after conservative measures and impairs activities of daily living [2,8]. It does not entail repeated dural puncture; it involves the epidural injection of autologous blood, and it has a distinct mechanism, evidence base and risk profile. Current multisociety guidance recognises that GONB may be offered in selected settings but continues to emphasise EBP for persistent, function-limiting symptoms [2]. Because no EBP was documented in this cohort, this study could not determine whether GONB reduces the use of EBP, functions as a bridge to it, is equivalent to it, or should occupy any particular position in the treatment algorithm. GONB is therefore best described here as an investigational symptomatic option rather than an established alternative to EBP.

The exploratory penalised analysis identified baseline pain intensity and orthopaedic surgery as factors associated with the receipt of repeated blocks, and the penalised estimates were considerably more stable than those of the original overfitted model. They nevertheless remain imprecise, are derived from a small single-centre cohort, and describe an outcome mediated by clinician decisions rather than an objective endpoint. They cannot be used for individualised treatment planning, and prospective validation with a prespecified retreatment rule would be required before any such use. Future studies should standardise the block technique and the co-interventions, define retreatment criteria in advance, systematically ascertain adverse events, record functional outcomes and specify a rescue EBP pathway.

## 5. Limitations

This study has several important limitations. Its retrospective, single-centre and uncontrolled design precluded causal inferences and could not separate any change related to GONB from spontaneous recovery, regression to the mean, concomitant treatment or the effect of repeat blocks. Because the repeated-block group was defined by the response at 24 h, comparisons between the groups were affected by indication and response bias and are presented as descriptive trajectories. Nine patients were excluded because of incomplete records, which may have introduced selection bias. Conservative regimens and post-block analgesic exposure were heterogeneous and were not documented in a form that allowed for standardisation. The sample was small and, with 24 events, the originally planned multivariable model could not be estimated in a stable manner; the penalised replacement was exploratory, was not internally validated and remained imprecise. The number of dural puncture attempts could not be retrieved. All blocks were performed by a single specialist using a single-level landmark-guided technique, which limits generalisability to other operators and to ultrasound-guided or bilevel approaches. Functional recovery, mobilisation, discharge timing, analgesic consumption and patient satisfaction were unavailable. Adverse events were ascertained retrospectively and may have been under-recorded. Systematic NRS follow-up ended on day 7, so neither later relapse nor the eventual course of the five patients with residual pain could be characterised. Finally, because no EBP was documented, no inference about the avoidance of EBP is possible.

This study also has strengths. All consecutive patients presenting during a clearly defined period were screened, and the study is reported in accordance with the STROBE statement [28]. The block technique was standardised and delivered by a single experienced operator; the NRS score was assessed at prespecified time points over seven days; the response-triggered rule for repeat sessions is reported transparently; and obstetric and non-obstetric surgical contexts are represented. These features make the dataset informative for describing clinical trajectories and for designing adequately controlled prospective studies, but they do not offset the absence of a comparator group.

## 6. Conclusions

In this retrospective cohort, NRS scores decreased during a seven-day management strategy comprising one or more bilateral GONB sessions with concomitant conservative care. Thirty-eight of 62 patients (61.3%) received a single block, and higher baseline pain intensity and orthopaedic surgery were associated with the receipt of repeated procedures in an exploratory penalised analysis. These findings are associative and hypothesis-generating: they do not establish that GONB caused the observed improvement, and they do not support the use of baseline pain or surgical context to individualise treatment. Prospective randomised studies with standardised co-interventions, prespecified retreatment criteria, systematic adverse-event ascertainment, functional outcomes and an appropriate control group are required. A direct comparison with EBP should be undertaken only where it is clinically and ethically appropriate, without implying equivalence to the established treatment.

## Figures and Tables

**Table 1:** Demographic characteristics and clinical features of the study cohort (*n* = 62).

Variable	*n* or Mean	% or ±SD
Sex, Female/Male	40/22	64.5%/35.5%
Age (years)	33.9	±15.1
Height (cm)	166.6	±8.5
Weight (kg)	69.6	±10.7
BMI (kg/m^2^)	25.1	±3.6
**Surgical Type**		
Caesarean section (C/S)	24	38.7%
Orthopaedic surgery	19	30.6%
Urological surgery	13	21.0%
General surgery	6	9.7%
**Number of GONB Sessions**		
1 block	38	61.3%
2 blocks	19	30.6%
3 blocks	4	6.5%
4 blocks	1	1.6%

BMI: body mass index; C/S: caesarean section; GONB: greater occipital nerve block; SD: standard deviation.

**Table 2:** Temporal changes in NRS pain scores (*n* = 62).

Time Point	*n*	Median	IQR (Q1–Q3)	Min	Max	*p* *	r †
Pre-block	62	7.0	6.0–8.0	4	10	—	—
Day 1	62	3.5	2.0–4.75	0	8	<0.001	0.60
Day 2	62	2.0	1.0–3.75	0	8	<0.001	0.61
Day 3	62	1.0	0.0–2.75	0	7	<0.001	0.62
Day 4	62	0.0	0.0–2.0	0	7	<0.001	0.62
Day 7	62	0.0	0.0–0.0	0	5	<0.001	0.62

NRS: Numerical Rating Scale; IQR: interquartile range (Q1–Q3). * Bonferroni-corrected Wilcoxon signed-rank test, each time point compared with the pre-block value. † Matched-pairs rank correlation effect size (r = Z/√2n). Overall Friedman test: χ^2^ = 262.6, df = 5, *p* < 0.001; Kendall’s W = 0.847. Values from day 2 onwards reflect management that included repeat blocks in 24 patients and cannot be attributed to the initial block.

**Table 3:** Distribution of pain severity categories over time (*n* = 62).

Time Point	Absent–Mild (NRS 0–3) *n* (%)	Moderate (NRS 4–7) *n* (%)	Severe (NRS 8–10) *n* (%)
Pre-block	—	36 (58.1%)	26 (41.9%)
Day 1	31 (50.0%)	26 (41.9%)	5 (8.1%)
Day 2	46 (74.2%)	14 (22.6%)	2 (3.2%)
Day 3	53 (85.5%)	9 (14.5%)	0 (0.0%)
Day 4	56 (90.3%)	6 (9.7%)	0 (0.0%)
Day 7	57 (91.9%)	5 (8.1%)	0 (0.0%)

Values are presented as *n* (%). NRS: Numerical Rating Scale.

**Table 4:** Comparison of patients receiving single versus repeated GONB.

Variable	Single Block (*n* = 38)	Repeated Block (*n* = 24)	*p*	Effect Size
**Demographic characteristics**				
Age (years)	27.0 (23.2–34.5)	30.5 (23.8–47.8)	0.304	r = 0.16
BMI (kg/m^2^)	24.9 (23.0–26.6)	25.5 (21.6–27.4)	0.960	r = 0.01
Female sex, *n* (%)	25 (65.8%)	15 (62.5%)	0.792	φ = 0.03
**Surgical category (overall)**			0.044 (exact 0.042)	Cramér’s V = 0.361
Caesarean section	19 (50.0%)	5 (20.8%)	—	—
Orthopaedic surgery	7 (18.4%)	12 (50.0%)	—	—
Urological surgery	8 (21.1%)	5 (20.8%)	—	—
General surgery	4 (10.5%)	2 (8.3%)	—	—
**Pain intensity (NRS)**				
Pre-block	6.0 (6.0–7.0)	8.0 (7.0–9.0)	<0.001	r = 0.62
Day 1	3.0 (2.0–3.0)	4.0 (4.0–5.25)	<0.001	r = 0.72
Day 2	2.0 (0.0–3.0)	2.5 (2.0–4.0)	0.028	r = 0.33
Day 3	1.0 (0.0–2.0)	2.0 (1.0–3.0)	0.048	r = 0.29
Day 4	0.0 (0.0–1.0)	1.0 (0.0–2.0)	0.026	r = 0.31
Day 7	0.0 (0.0–0.0)	0.0 (0.0–1.0)	0.069	r = 0.21

Values are median (IQR: Q1–Q3) or *n* (%). Group membership was determined by the response at 24 h and by subsequent clinical decisions, so all comparisons in this table are descriptive. Continuous variables: Mann–Whitney U test with the rank-biserial correlation r as the effect size. Sex: chi-square test with φ. Surgical category: overall chi-square test (χ^2^ = 8.093, df = 3) with Cramér’s V; because two expected counts were below five, a Monte Carlo exact *p* value (200,000 permutations) is also reported. No category-specific post hoc comparison was performed and none should be inferred from the cell percentages. BMI: body mass index; IQR: interquartile range; NRS: Numerical Rating Scale.

**Table 5:** Comparison of demographic, procedural, and NRS-based pain outcomes between caesarean and non-caesarean subgroups.

Variable	C/S Group (*n* = 24)	Non-C/S Group (*n* = 38)	*p*	Effect Size
Age (years)	27.0 (24.8–32.0)	30.0 (21.5–55.2)	0.325	r = 0.15
BMI (kg/m^2^)	25.0 (23.5–26.7)	23.7 (21.8–27.6)	0.251	r = 0.18
Female sex, *n* (%)	24 (100.0%)	16 (42.1%)	not tested *	—
Pre-block NRS	7.0 (6.0–8.0)	7.0 (6.0–9.0)	0.431	r = 0.12
Number of blocks	1.0 (1.0–1.0)	1.5 (1.0–2.0)	0.015	r = 0.32
Repeated GONB (≥2 sessions), *n* (%)	5 (20.8%)	19 (50.0%)	0.032 †	—
Adequate analgesia on day 1 (NRS < 4), *n* (%)	14 (58.3%)	17 (44.7%)	0.435 †	—
Day 1 NRS	3.0 (3.0–4.0)	4.0 (2.0–5.0)	0.780	r = 0.04
Day 2 NRS	2.0 (1.0–3.25)	2.0 (0.25–3.75)	0.612	r = 0.08
Day 3 NRS	1.0 (0.0–3.0)	1.0 (0.0–2.0)	0.982	r < 0.01
Day 4 NRS	0.0 (0.0–2.25)	0.0 (0.0–2.0)	0.838	r = 0.03
Day 7 NRS	0.0 (0.0–0.0)	0.0 (0.0–0.75)	0.743	r = 0.04
Absent-to-mild pain on day 7, *n* (%)	22 (91.7%)	35 (92.1%)	1.000 †	—
NRS trajectory (Friedman)	χ^2^ = 103.4, *p* < 0.001 (W = 0.862)	χ^2^ = 159.6, *p* < 0.001 (W = 0.840)	—	—

Values are median (IQR: Q1–Q3) or *n* (%). * Sex was not compared because all caesarean patients were women; surgical category and sex are structurally confounded in this cohort. † Fisher exact test. Continuous variables: Mann–Whitney U test with the rank-biserial correlation r as the effect size. Between-subgroup NRS comparisons were non-significant at every follow-up assessment (all *p* ≥ 0.61), consistent with the statement in the text. All analyses in this table are exploratory and are confounded by response-based treatment decisions. C/S: caesarean section; GONB: greater occipital nerve block; IQR: interquartile range; NRS: Numerical Rating Scale.

**Table 6:** Exploratory Firth penalised logistic regression for the receipt of repeated GONB (≥2 sessions).

Variable	OR	95% CI (Profile Likelihood)	*p*
Baseline NRS (per 1-point increase)	2.41	1.55–4.15	<0.001
Orthopaedic surgery (vs. all other categories)	5.02	1.36–21.56	0.015

Outcome: receipt of repeated GONB (≥2 sessions) versus a single session; *n* = 62, 24 events, 12 events per variable. Estimates were obtained by Firth’s penalised maximum likelihood; 95% confidence intervals are profile-likelihood intervals and *p* values are penalised likelihood-ratio tests [32,33]. The model was prespecified and is exploratory: it is reported for hypothesis generation, not as a prediction model, and no internal validation or calibration assessment was performed. Receipt of a repeated block is a clinician-mediated outcome determined after baseline. CI: confidence interval; GONB: greater occipital nerve block; NRS: Numerical Rating Scale; OR: odds ratio.

## Data Availability

The data and materials are available from the corresponding author upon reasonable request and are subject to ethical review.

## Abbreviations

The following abbreviations are used in this manuscript:

PDPH	Post-dural puncture headache
EBP	Epidural blood patch
GONB	Greater occipital nerve block
NRS	Numerical Rating Scale
CSF	Cerebrospinal fluid
NSAIDs	Non-steroidal anti-inflammatory drugs
GON	Greater occipital nerve
STROBE	Strengthening the Reporting of Observational Studies in Epidemiology
BMI	Body mass index
C/S	Caesarean section
ICHD-3	International Classification of Headache Disorders, 3rd edition
IQR	Interquartile range
CI	Confidence interval
OR	Odds ratio
